# Supplementary material for: Caspar modulates primordial germ cell fate both in an Oskar-dependent and Oskar-independent manner
Source: Biol Open. 2025 Jul 28;14(7):bio062119. doi: 10.1242/bio.062119 (PMC12352277; doi:10.1242/bio.062119)
Supplement: Supplementary information [file biolopen-14-062119-s1.pdf]

A

Number of embryos (n) imaged and Average number of PGCs (Av) for Fig. 1, panels B &C

Fig. 1 Panel B	n	Av		Fig. 1 Panel C	n	Av
B1	18	30.5		C1	20	20.4
B2	32	7.3		C2	51	8.8
B3	18	26.8		C3	14	19.7
B4	18	6.6		C4	16	5.9
B5	17	33		C5	19	26.3

B

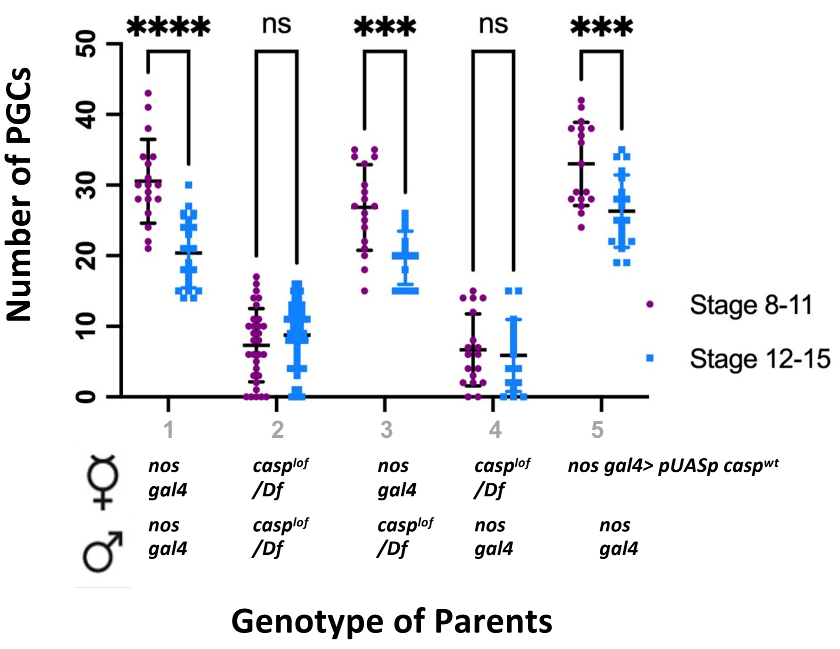

Fig. S1. Stage-wise comparison of defects in PGC migration in *casp^lof* embryos.

Data from Figure 1 (panels B & C) is represented with identical genotypes paired together for stages 8-11 & 12-15. The parental genotype is listed on the X-axis with total number of PGCs plotted along the Y-axis. Each point on the graph represents one embryo, with 14-50 embryos represented (See Fig. S1A) per genotype, per stage.

The graph highlights (a) The normal drop of PGCs with the progress in development for embryos laid by wild-type mothers– Genotypes #1 & #3 (b) The reduced number of PGCs for embryos laid by *casp^lof* mothers (genotypes #2 & #5), and the interesting fact that these PGCs do not show a drop in numbers between stages 8-11 & 12-15 (c) The higher than normal PGCs for embryos laid by *casp^OE* mothers (genotype #5), and their drop in numbers. Unpaired t-test, (****) p<0.0001; ns, insignificant.

Biology Open • Supplementary information

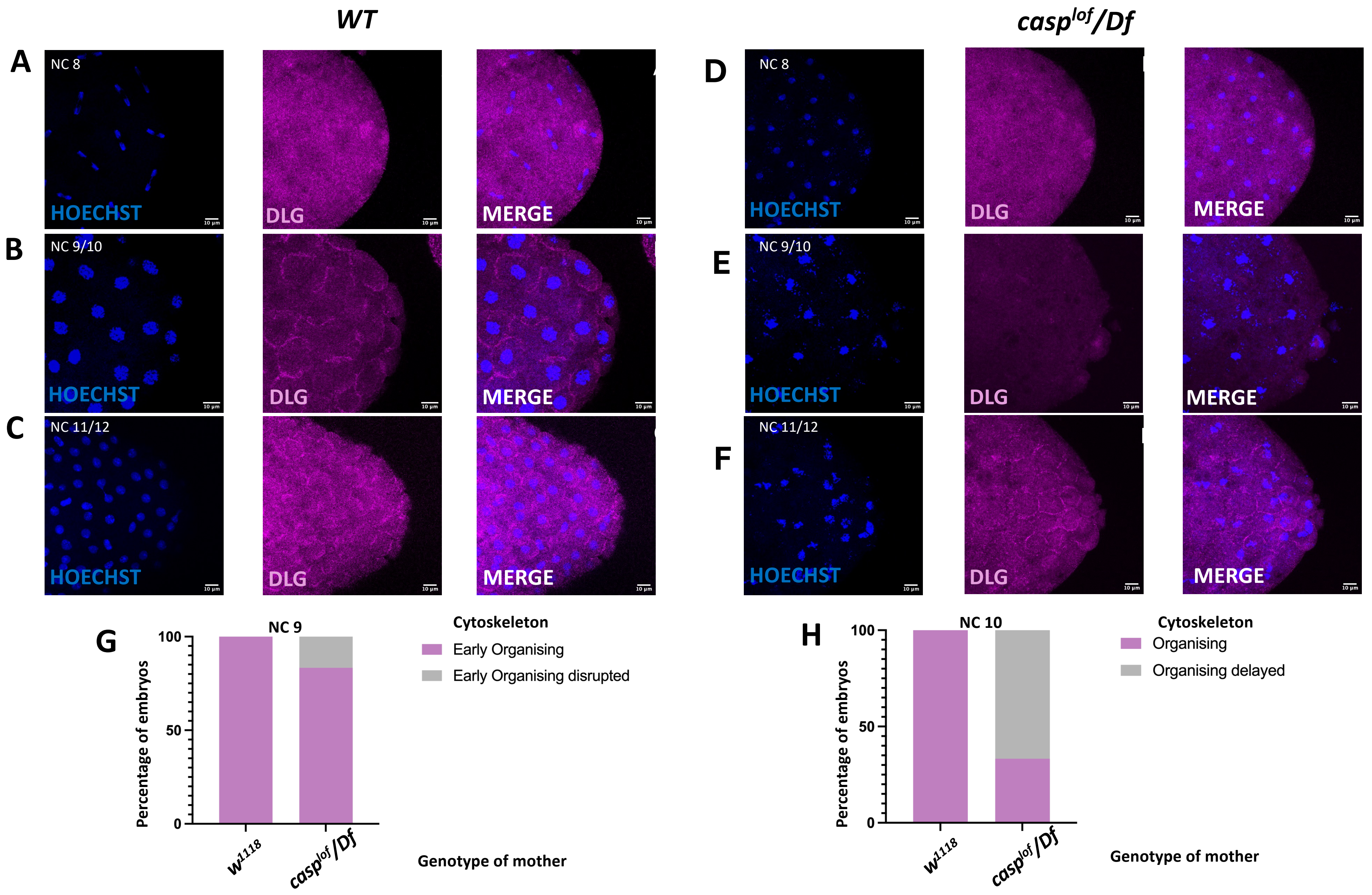

Fig S2. Somatic compartmentalisation is initiated at the same time as pole cell budding and is regulated by Casp.

(A, D) Nuclear cycle 8, nuclear cycle 9/10, **(B, E)** and nuclear cycle 10/11 **(C,F)** in w^{1118} (panels A-C) in *casp^{lof}/Df* (panels D-F) embryos immunostained with Disc Large antibodies and Hoechst DNA dye, showing delay in somatic compartmentalization. **(B, E)** NC 9-10 (panel G) for w^{1118} and *casp^{lof}/Df* (panel H) embryos; Bar graphs showing the percentage of embryos with cytoskeletal defects.

Biology Open • Supplementary information
